# From contact coverage to effective coverage of community care for patients with severe mental disorders: a real-world investigation from Italy. Methodology and results from the QUADIM project

**DOI:** 10.1192/j.eurpsy.2023.228

**Published:** 2023-07-19

**Authors:** M. Monzio Compagnoni, G. Corrao, A. Barbato, B. D’Avanzo, T. Di Fiandra, F. Carle, L. Ferrara, V. D. Tozzi, A. Gaddini, A. Saponaro, S. Scondotto, D. Chisholm, A. Lora

**Affiliations:** 1 Department of Statistics and Quantitative Methods; 2 National Centre for Healthcare Research and Pharmacoepidemiology, University of Milano-Bicocca; 3 Unit for Quality of Care and Rights Promotion in Mental Health, Istituto di Ricerche Farmacologiche Mario IRCCS, Milan; 4 Previously General Directorate for Health Prevention, Italian Ministry of Health, Rome; 5 Center of Epidemiology and Biostatistics; 6 National Centre for Healthcare Research and Pharmacoepidemiology, Polytechnic University of Marche, Ancona; 7 Centre of Research on Health and Social Care Management, SDA Bocconi School of Management (Bocconi University), Milan; 8 Agency for Public Health, Lazio Region, Rome; 9 General Directorate of Health and Social Policies, Emilia-Romagna Region, Bologna; 10 Department of Health Services and Epidemiological Observatory, Regional Health Authority, Sicily Region, Palermo, Italy; 11 Department of Mental Health and Substance Abuse, World Health Organization, Geneva, Switzerland; 12 Department of Mental Health and Addiction Services, ASST Lecco, Lecco, Italy

## Abstract

**Introduction:**

The evaluation of healthcare pathways must be considered of fundamental importance. The quality of care provided to patients with severe mental disorders (SMD) does not correspond to the standards set by the recommendations. Therefore, measures such as the real coverage rate of psychiatric patients’ needs (*contact coverage*), by comparing epidemiological prevalence rates and the number of patients receiving adequate care, could be a valuable resource for implementing the transition to community mental health. However, simple assessment and reporting of rates of contact with mental healthcare potentially overestimate the full expected health benefits of services. Therefore, in addition to monitor the coverage rate achieved by the services, the evaluation of the *effectiveness* of the care provided (*effective coverage*) [De Silva *et al*. Int J Epidemiol 2014;43(2):341–53] is also of relevant importance.

**Objectives:**

To measure the gap between contact and *effective coverage* of mental healthcare, i.e., the *effectiveness* of interventions provided by services for the treatment of SMD in preventing an exacerbation of psychiatric symptoms.

**Methods:**

Data were retrieved from Healthcare Utilization databases of four Italian Regions (Lombardy, Emilia-Romagna, Lazio, Sicily). 45,761 newly taken-in-care cases of depression, schizophrenia, bipolar, and personality disorder were included. A variant of the self-controlled case series method was used to estimate the incidence rate ratio (IRR) for the relationship between exposure (use of different types of mental healthcare such as pharmacotherapy, generic contacts with the outpatient service, psychosocial interventions, and psychotherapies) and relapse episodes (mental illness emergency hospital admissions).

**Results:**

11,500 relapses occurred. Relapse risk was reduced (**Figure**) during periods covered by (i) psychotherapy for patients with depression (IRR 0.67; 95% CI, 0.49 to 0.91) and bipolar disorder (0.64; 0.29 to 0.99); (ii) psychosocial interventions for those with depression (0.74; 0.56 to 0.98), schizophrenia (0.83; 0.68 to 0.99) and bipolar disorder (0.55; 0.36 to 0.84), (iii) pharmacotherapy for those with schizophrenia (0.58; 0.49 to 0.69), and bipolar disorder (0.59; 0.44 to 0.78). Coverage with generic mental healthcare, in the absence of psychosocial/psychotherapeutic interventions, did not affect the risk of relapse.

**Image:**

**Figure:**
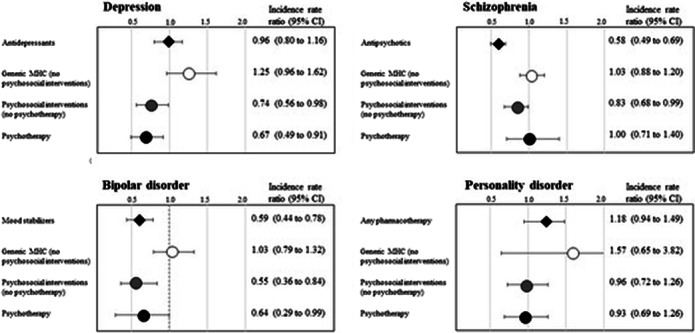

**Conclusions:**

Psychosocial interventions, psychotherapies and specific pharmacotherapies can be considered particularly effective in treating patients with bipolar, depressive, and schizophrenic disorders. This study ascertained the gap between utilization of mental healthcare and *effective coverage*, showing that *real-world* data can represent a useful resource to monitor mental healthcare paths and to assess the effectiveness of a mental health system.

**Disclosure of Interest:**

None Declared

